# Epitheloid Myxofibrosarcoma of the Parotid Gland

**DOI:** 10.1155/2011/641621

**Published:** 2011-09-13

**Authors:** B. Srinivasan, M. Ethunandan, K. Hussain, V. Ilankovan

**Affiliations:** ^1^Department of Oral and Maxillofacial Surgery, St Richard's Hospital, Spitalfield Lane, Chichester PO19 6SE, UK; ^2^St Richard's Hospital, Spitafield Lane, Chichester, UK; ^3^Department of Histopathology, Poole Hospital NHS Trust, Longfleet Road, Poole, Dorset BH15 2JB, UK

## Abstract

Myxofibrosarcoma has been recently described as a distinct histological entity and commonly affects the extremities and trunk of the elderly. It is, however, rare in the head and neck (2.8%) region, and we are unaware of any reports of it presenting as a parotid mass. The epitheloid variant accounts for less than 3% of myxofibrosarcomas. We report a case of a 78/F presenting with an epitheloid myxofibrosarcoma in the parotid gland. The clinical presentation was of a parotid lump of 9-month duration, and the diagnosis was made following thorough histological assessment. We present what we believe to be the first reported case of a (epitheloid) myxofibrosarcoma affecting the parotid gland and highlight its diagnostic features and outcome of treatment.

## 1. Introduction

Myxofibrosarcoma has been recently described as a distinct histological entity and commonly affects the extremities and trunk of the elderly [1–4]. It is, however, rare in the head and neck region, and we are unaware of any reports of this presenting as a parotid mass. We report a case of myxofibrosarcoma presenting as a parotid lump and highlight the clinical course and histological features of this lesion. 

## 2. Report of Case

**Figure 1:**
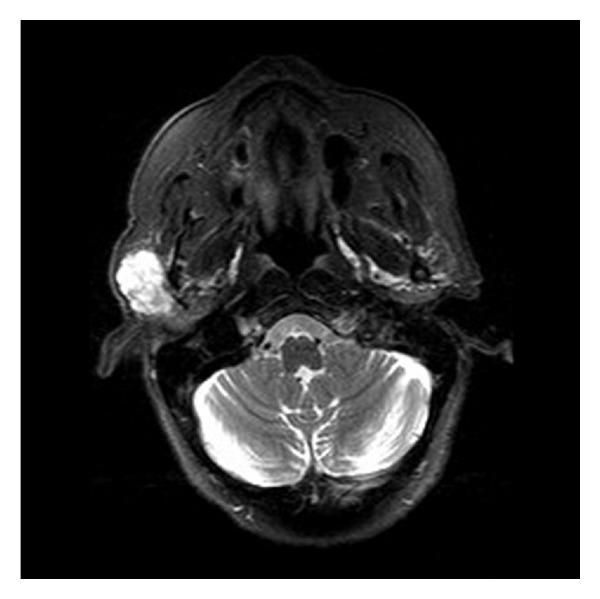
MRI demonstrating tumour in the right parotid gland.

**Figure 2:**
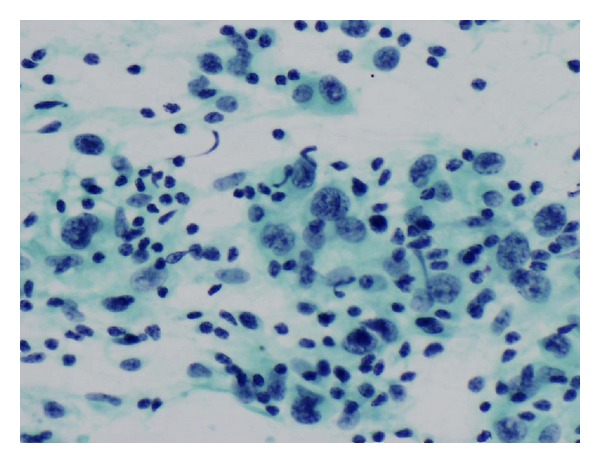
FNAC demonstrating undifferentiated pleomorphic tumour cells in addition to lymphocytes and histiocytes (Pap. ×400).

A 78-year-old female presented with a lump in the right parotid region of a nine-month duration. Clinical examination revealed a 4 × 3 cm mildly tender, mobile mass with normal facial nerve function. There was no palpable cervical lymphadenopathy. An MRI scan revealed a 3.8 cm well marginated mass largely confined to the superficial lobe of the parotid and involving the lateral edge of the deep lobe, (Figure 1). Fine needle aspiration cytology demonstrated undifferentiated pleomorphic malignant cells with frequent lymphocytes and histiocytes present in the background (Figure 2). A tentative diagnosis of a malignant mesenchymal tumour (sarcoma) was made and the patient underwent a total conservative parotidectomy and a selective level I–III neck dissection. The tumour was directly overlying the main trunk and upper branches of the facial nerve. A few peripheral branches were involved due to direct involvement by the tumour. The final histology was reported as a high-grade epitheloid myxofibrosarcoma of the parotid, with tumour extending to the resection margin and two of the 13 lymph nodes harvested demonstrated metastatic spread (level II). The patient received postoperative radiotherapy to the parotid bed and neck (60 Gray/30 fractions). The patient was followed up for 18 months with no evidence of local, regional, or distant metastasis, but unfortunately died of motor neurone disease 24 months after initial presentation. An autopsy was not performed.

## 3. Histology

### 3.1. Macroscopic Features

 The parotid gland (60 × 40 × 25 mm) was weighing 28 grams. One pole contains a multinodular tumor nodule (36 × 24 × 24 mm) with a gelatinous myxoid cut surface. A second irregular fragment of parotid tissue measuring (75 × 17 × 15 mm) was weighing 6.5 grams. 

### 3.2. Microscopic Features

**Figure 3:**
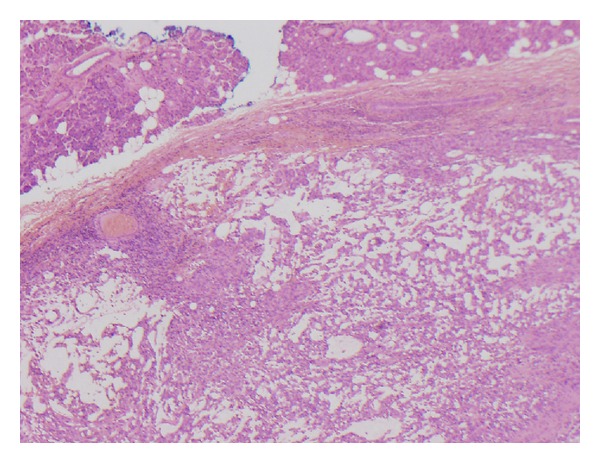
Tumour cells arranged in a loose lacelike pattern in a myxoid stroma. The margin is well defined in this area and normal salivary tissue is present in the upper right corner (H&E ×20).

**Figure 4:**
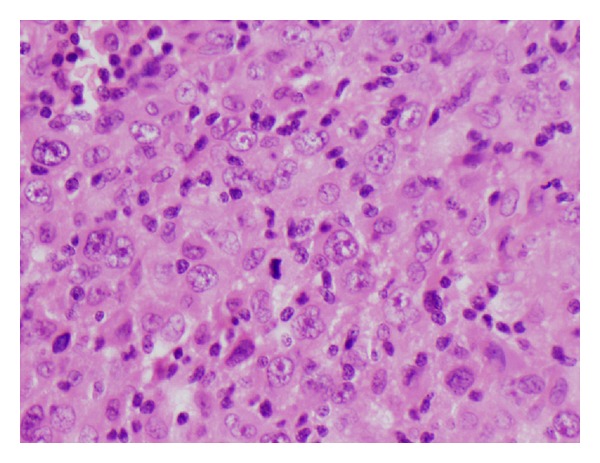
Pleomorphic epitheloid cells having vesicular nuclei and prominent nucleoli. A mitosis is present at the centre.

**Figure 5:**
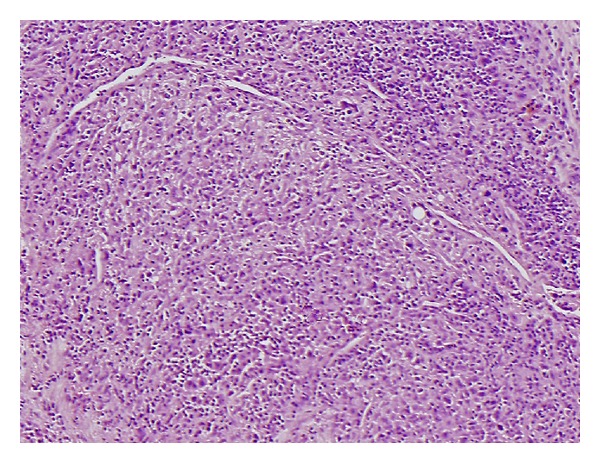
Tumour cell arranged along curvilinear blood vessels (H&E ×100).

**Figure 6:**
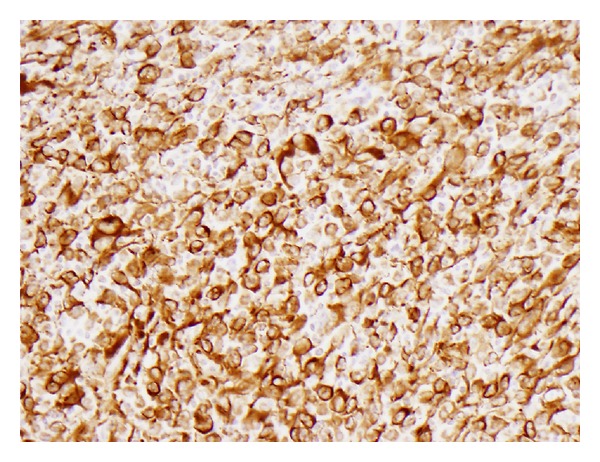
Immunohistochemistry demonstrating tumour cells staining for vimentin (Vimentin×200).

The tumour was well circumscribed and had well-defined outer margin with a pseudocapsule in places (Figure 3). It was composed of epitheloid/spindle-shaped pleomorphic tumour cells with abundant eosinophilic cytoplasm showing frequent mitotic figures (15 mitosis/10 HPF) and abnormal mitoses (Figure 4). Frequent lymphocytes and reactive histiocytes were present in the background. Multiple foci of prominent myxoid change were present. Spaces filled with alcian blue-positive mucin separated cordlike arrangements of cells resulting in a lacelike pattern. In some places, the tumour formed solid sheets with little mucin and in other it was notably arranged along scanty curvilinear capillaries in the myxoid background (Figure 5). Remnants of salivary tissue were present within the tumour. Immunohistochemistry demonstrated that the tumour cells stained cells with vimentin (Figure 6). The tumor cells were not immunoreactive with Cam 5.2, 34BE12 and S100, MART1, microphthalmia transcription factor (MITF), and CD34 were negative. P63 showed equivocal staining. Taking into account the morphology and immunophenotype, a diagnosis of high-grade epitheloid myxofibrosarcoma was made. Two of the eight lymph nodes in level 2 contained metastatic tumour but three lymph nodes in level 3 and the lymph node adjacent to the submandibular salivary gland showed reactive changes only.

## 4. Discussion

Myxofibrosarcoma, often reported in the past as the myxoid variant of the malignant fibrous histiocytoma, was initially suggested as a distinct histological entity by Angervall et al. in 1977 [1] and subsequently more widely recognized following its inclusion in the WHO classification in 2002 [2]. The lesions do not demonstrate any obvious sex predilection and often affect the elderly, with a mean age of 60 years (range of 16–91 years) [3, 4]. It frequently presents as a slowly enlarging painless mass of weeks to months duration, though 75% of them were less than 18 months duration [3]. It often affects the extremities and trunk, and involvement of the head and neck region (2.8%) is uncommon [3, 4]. Our case was relatively typical in terms of age and sex, but we are unaware of any previous reports of the lesion affecting the parotid/salivary gland. Epitheloid myxofibrosarcoma is a rare variant of myxofibrosarcoma and accounts for less than 3% of myxofibrosarcomas [5]. The definitive diagnosis is often made following thorough histological assessment, often supplemented by IHC, though the cytological features might be suggestive [1–8]. Cytology often demonstrates large, bizarre, multinucleated giant cells with abundant eosinophilic and well-delineated cytoplasm and irregular-shaped and prominent nuclei. Histologically, the lesions demonstrate a broad spectrum of cellularity, pleomorphism, and proliferative activity and exhibit at least a focal area of myxoid matrix. Typically, elongated curvilinear capillaries with perivascular tumour cell condensation are seen in the myxoid areas. High-grade tumours demonstrate solid, hyper cellular areas with conspicuous cellular pleomorphism, numerous, often atypical mitosis, and confluent areas of hemorrhage and necrosis. In addition, there is often a heavy inflammatory infiltrate, mainly composed of lymphocytes and plasma cells. The characteristic tumour cells in the myxoid areas stain positively for vimentin, but do not stain for other markers. 

The mainstay of treatment is excision of the lesion with wide margins, supplemented if necessary by radiotherapy [9]. Local recurrence is not uncommon, with a reported frequency of 45–61%, with the lesion becoming progressively higher grade with repeated recurrences [4, 9]. Metastasis occurred in about 23% of cases and often affected the lungs, lymph nodes, and somatic soft tissues [4, 10, 11] Various clinicopathologic factors including the status of the surgical margins, grade of tumour, mitotic index and extent of necrosis have all been found to influence the prognosis [3, 4]. Epitheloid myxofibrosarcoma is considered to be more aggressive than high-grade myxofibrosarcoma with a 70% risk of local recurrence and 50% risk of metastasis [5]. 

The quality of primary surgery does not only improve local recurrence, but also translates into survival benefit, suggesting that thorough reexcision or adjuvant radiotherapy is warranted for lesions with positive margins [4, 9].
